# Supplementary material for: Competitiveness and Phylogenetic Relationship of Rhizobial Strains with Different Symbiotic Efficiency in Trifolium repens: Conversion of Parasitic into Non-Parasitic Rhizobia by Natural Symbiotic Gene Transfer
Source: Biology (Basel). 2023 Feb 3;12(2):243. doi: 10.3390/biology12020243 (PMC9953144; doi:10.3390/biology12020243)
Supplement: Supplementary file 1 [file biology-12-00243-s001.zip › Figure S5.pdf]

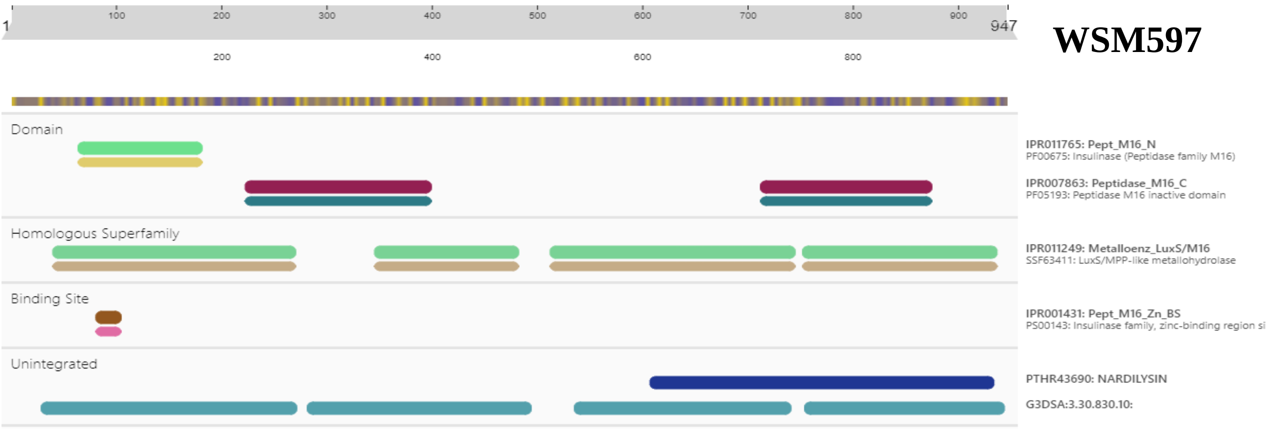
WSM597
1
100
200
300
400
500
600
700
800
900
947
200
400
600
800
Domain
Homologous Superfamily
Binding Site
Unintegrated
IPR011765: Pept_M16_N
PF00675: Insulinase (Peptidase family M16)
IPR007863: Peptidase_M16_C
PF05193: Peptidase M16 inactive domain
IPR011249: Metalloenz_LuxS/M16
SSF63411: LuxS/MPP-like metallohydrolase
IPR001431: Pept_M16_Zn_BS
PTHR43690: NARDILYSIN
G3DSA:3.30.830.10:

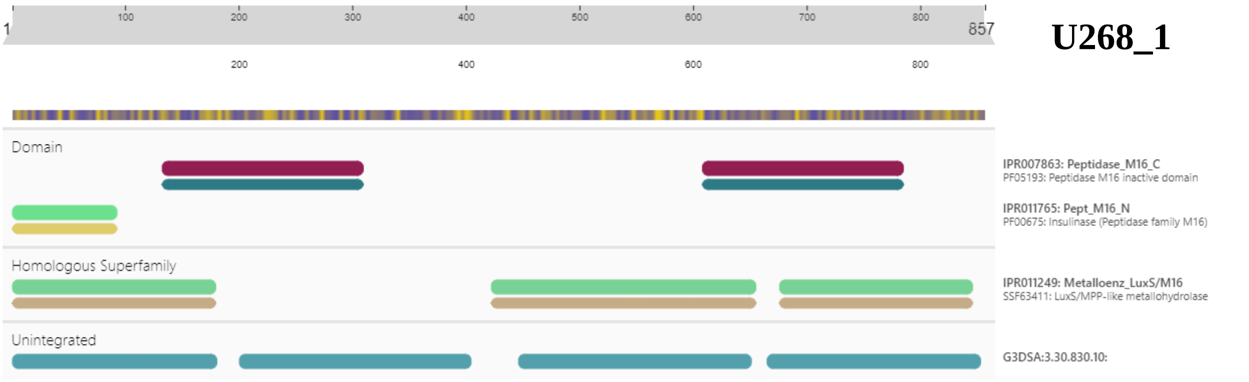
U268_1
1
100
200
300
400
500
600
700
800
857
200
400
600
800
Domain
Homologous Superfamily
Unintegrated
IPR007863: Peptidase_M16_C
PF05193: Peptidase M16 inactive domain
IPR011765: Pept_M16_N
PF00675: Insulinase (Peptidase family M16)
IPR011249: Metalloenz_LuxS/M16
SSF63411: LuxS/MPP-like metallohydrolase
G3DSA:3.30.830.10:

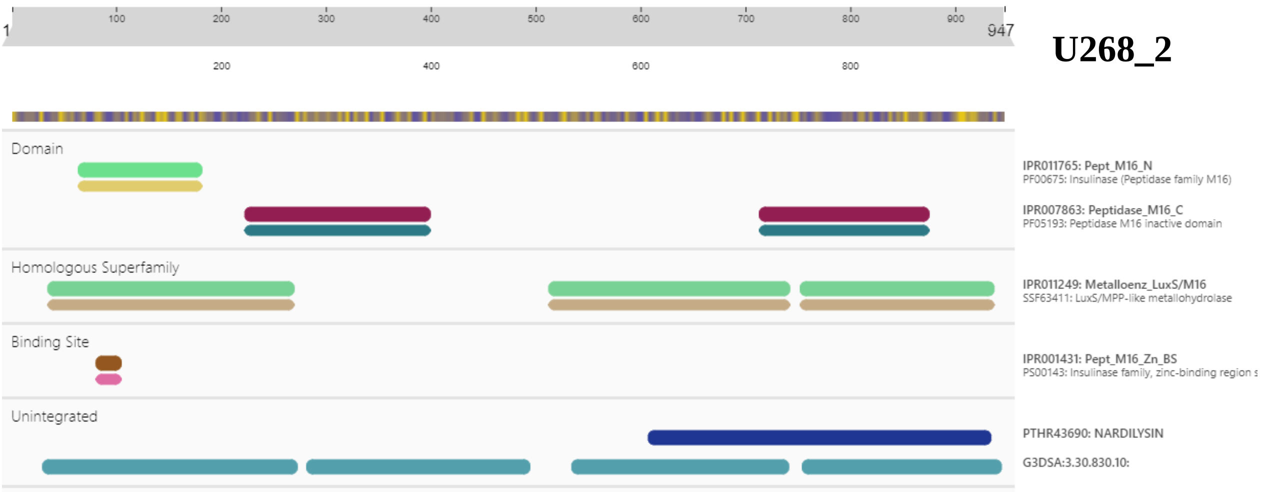
U268_2
1
100
200
300
400
500
600
700
800
900
947
200
400
600
800
Domain
Homologous Superfamily
Binding Site
Unintegrated
IPR011765: Pept_M16_N
PF00675: Insulinase (Peptidase family M16)
IPR007863: Peptidase_M16_C
PF05193: Peptidase M16 inactive domain
IPR011249: Metalloenz_LuxS/M16
SSF63411: LuxS/MPP-like metallohydrolase
IPR001431: Pept_M16_Zn_BS
PTHR43690: NARDILYSIN
G3DSA:3.30.830.10:

U204_1

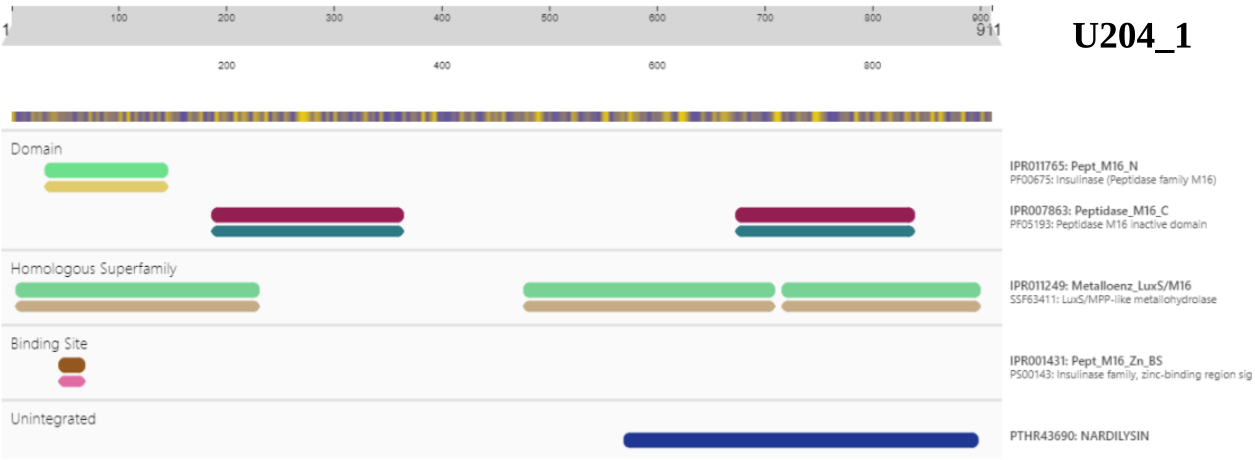

U204_2

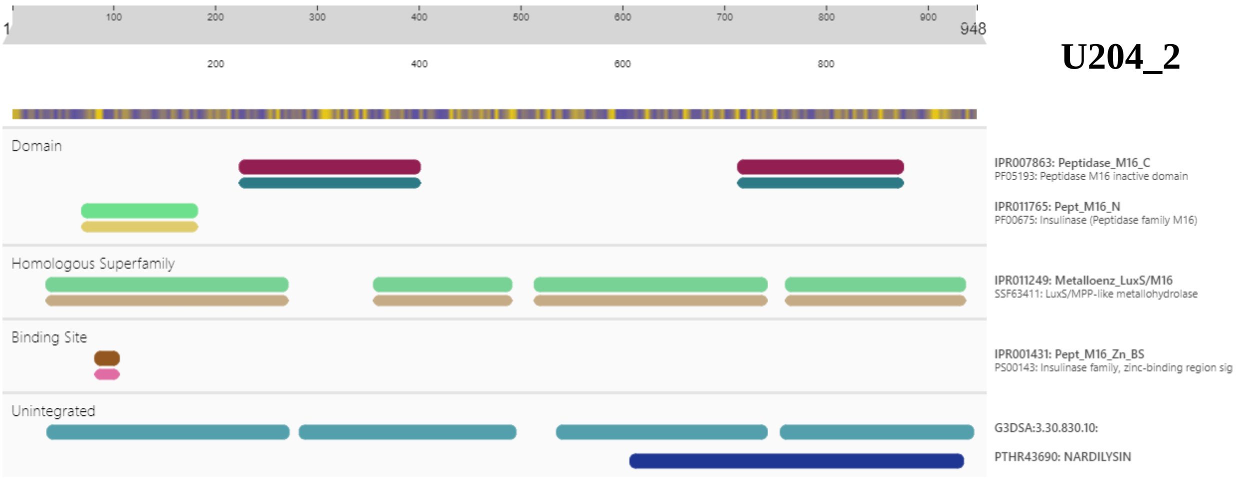

Figure S5. Graphical view of The InterPro search and protein classification of M16 peptidase sequences found in the genome of strains U268, U204 and WSM 597 using InterPro database. The InterPro entry was the aminoacidic sequence for the putative M16 peptidases. In the graphical view, matches to the InterPro databases and the relevant positions of these matches on the query sequence are displayed.
