# Supplementary figures and images for: Competitiveness and Phylogenetic Relationship of Rhizobial Strains with Different Symbiotic Efficiency in Trifolium repens: Conversion of Parasitic into Non-Parasitic Rhizobia by Natural Symbiotic Gene Transfer
Source: Biology (Basel). 2023 Feb 3;12(2):243. doi: 10.3390/biology12020243 (PMC9953144; doi:10.3390/biology12020243)

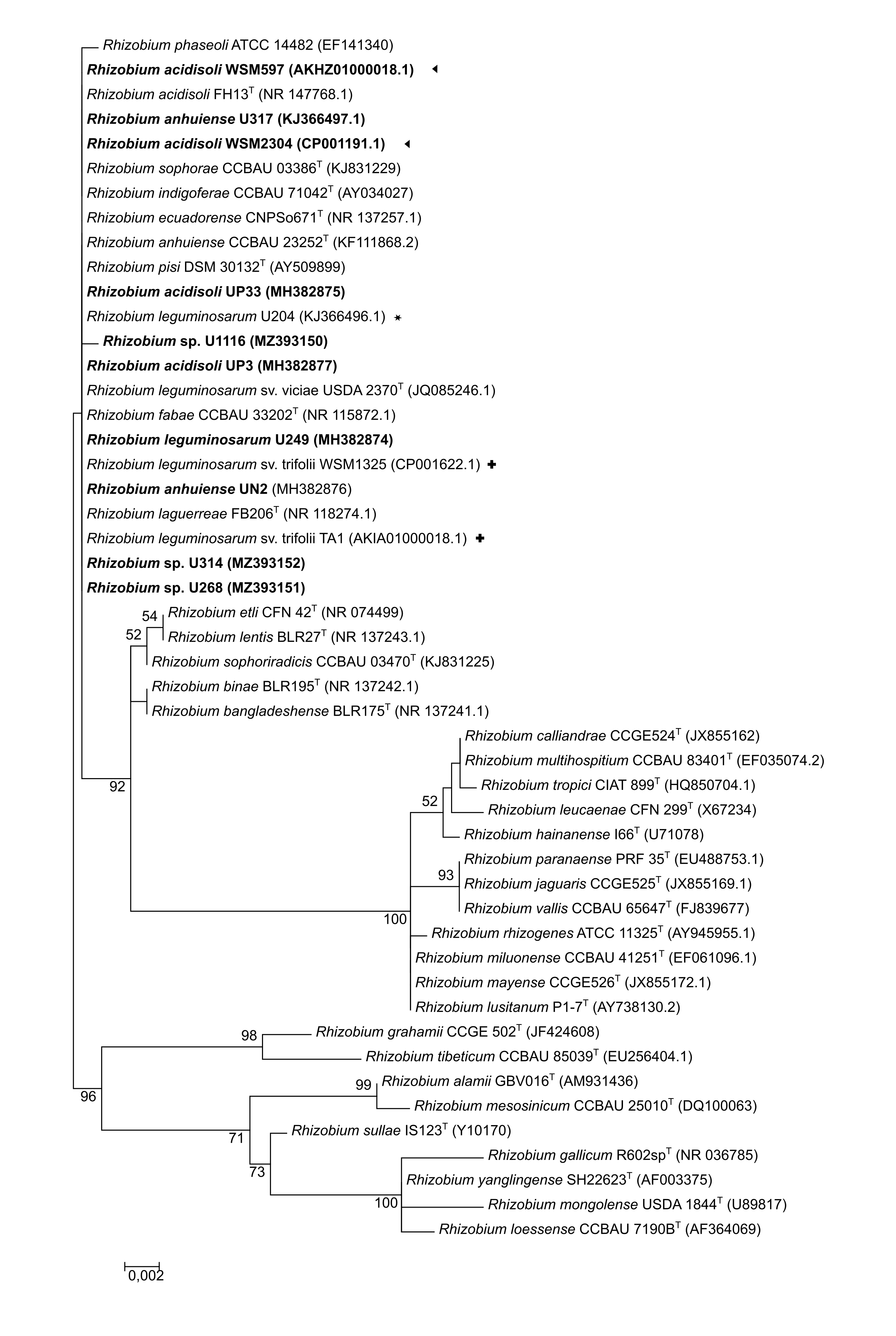

Supplement: Supplementary file 1 [file biology-12-00243-s001.zip › Figure S1.png]

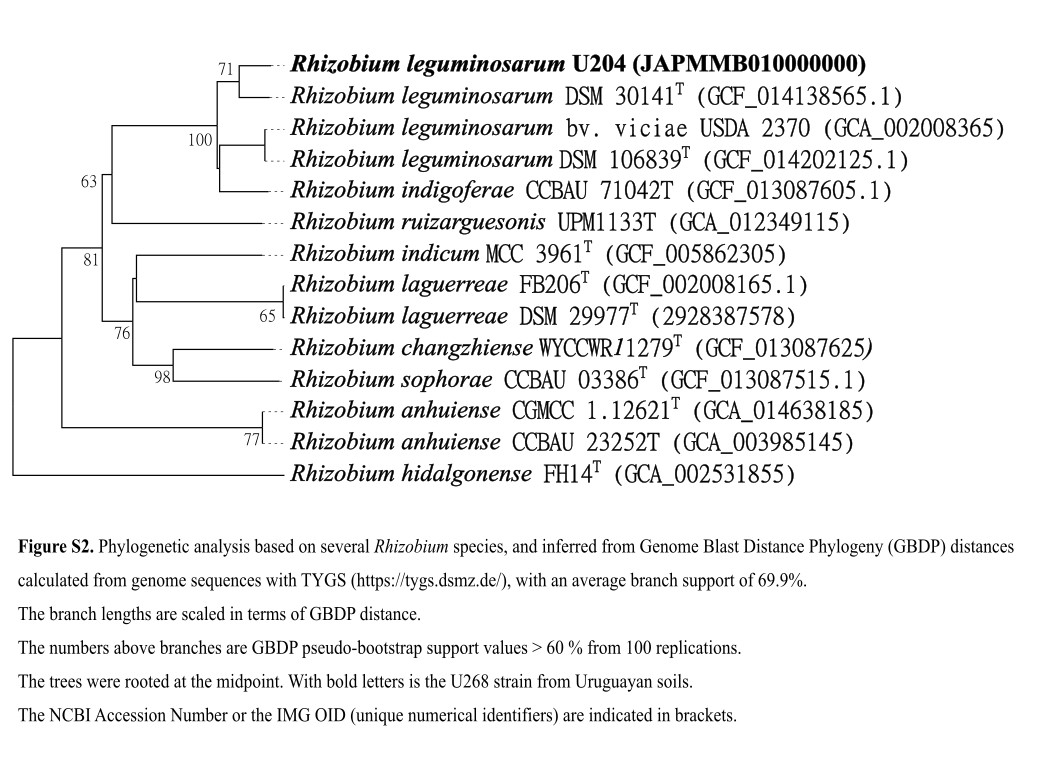

Supplement: Supplementary file 1 [file biology-12-00243-s001.zip › Figure S2.jpg]

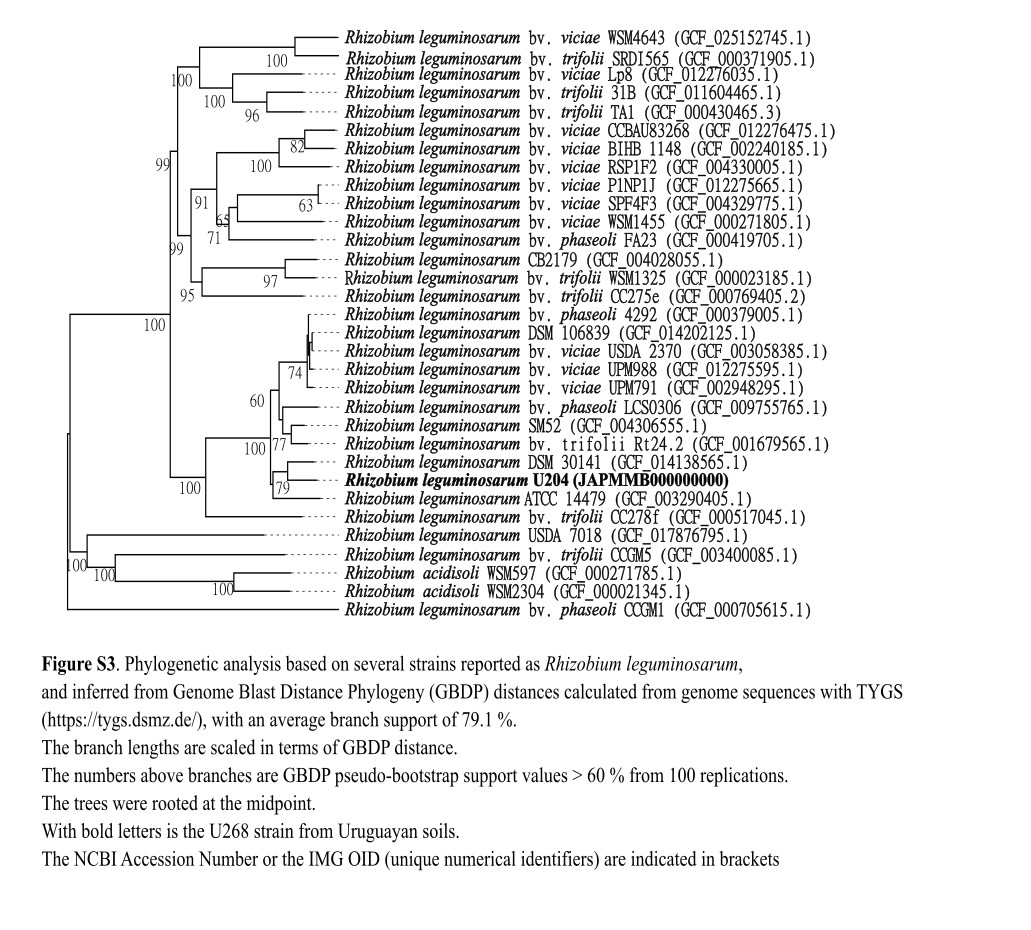

Supplement: Supplementary file 1 [file biology-12-00243-s001.zip › Figure S3.jpg]

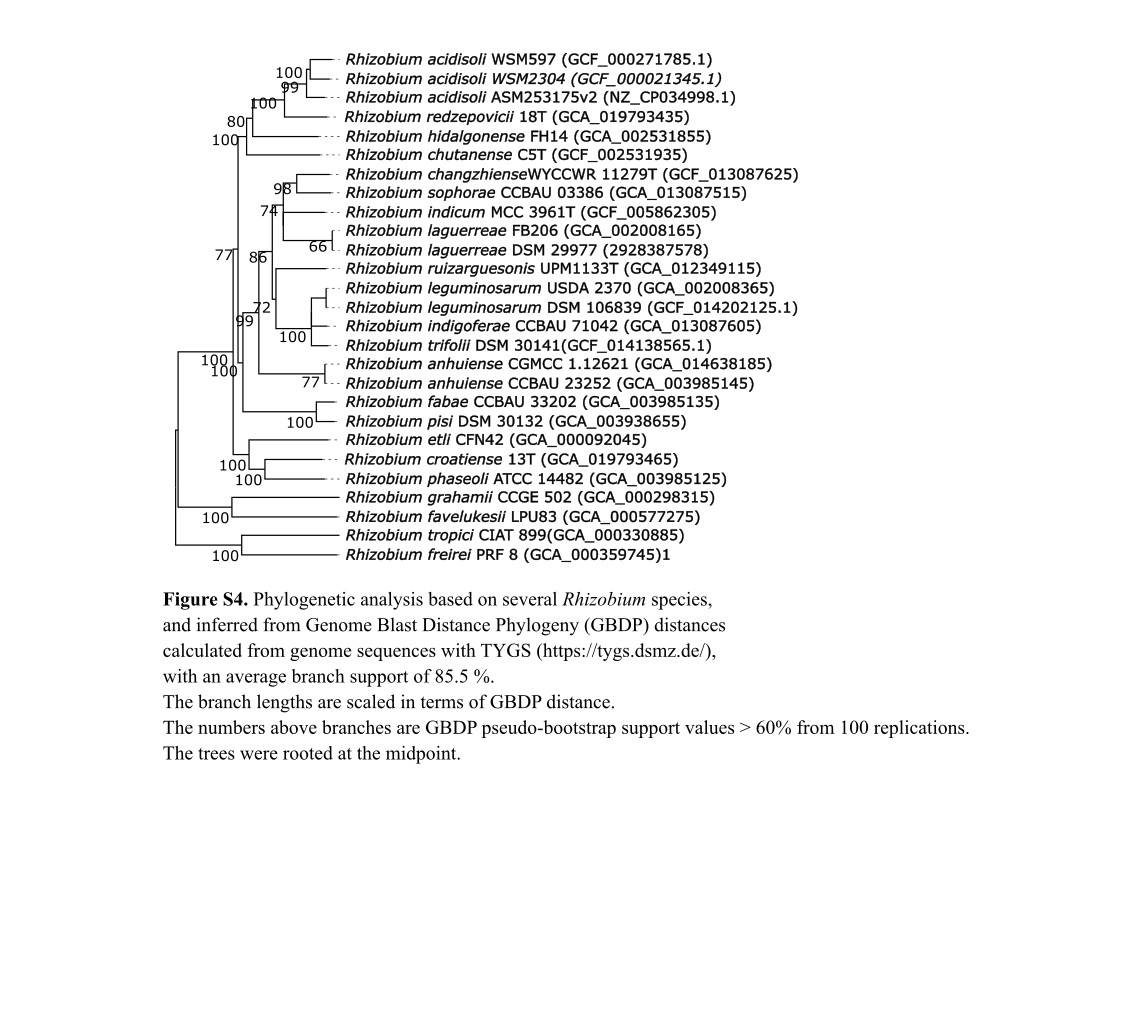

Supplement: Supplementary file 1 [file biology-12-00243-s001.zip › Figure S4.jpg]
